# Factors Associated With the Dilation of Perivascular Space in Healthy Elderly Subjects

**DOI:** 10.3389/fnagi.2021.624732

**Published:** 2021-03-26

**Authors:** Peiyu Huang, Zili Zhu, Ruiting Zhang, Xiao Wu, Yeerfan Jiaerken, Shuyue Wang, Wenke Yu, Hui Hong, Chunfeng Lian, Kaicheng Li, Qingze Zeng, Xiao Luo, Xiaopei Xu, Xinfeng Yu, Yunjun Yang, Minming Zhang

**Affiliations:** ^1^Department of Radiology, The Second Affiliated Hospital, Zhejiang University School of Medicine, Hangzhou, China; ^2^Department of Radiology, The First Affiliated Hospital of Wenzhou Medical University, Wenzhou, China; ^3^Department of Radiology, Biomedical Research Imaging Center, University of North Carolina at Chapel Hill, Chapel Hill, NC, United States

**Keywords:** perivascular space, aging, intracranial volume, artery diameter, white matter hyperintensity

## Abstract

**Background:** The dilation of perivascular space (PVS) has been widely used to reflect brain degeneration in clinical brain imaging studies. However, PVS characteristics exhibit large differences in healthy subjects. Such variations need to be better addressed before PVS can be used to reflect pathological changes. In the present study, we aim to investigate the potential influence of several related factors on PVS dilation in healthy elderly subjects.

**Methods:** One-hundred and three subjects (mean age = 59.5) were retrospectively included from a prospectively collected community cohort. Multi-modal high-resolution magnetic resonance imaging and cognitive assessments were performed on each subject. Machine-learning based segmentation methods were employed to quantify PVS volume and white matter hyperintensity (WMH) volume. Multiple regression analysis was performed to reveal the influence of demographic factors, vascular risk factors, intracranial volume (ICV), major brain artery diameters, and brain atrophy on PVS dilation.

**Results:** Multiple regression analysis showed that age was positively associated with the basal ganglia (BG) (standardized beta = 0.227, *p* = 0.027) and deep white matter (standardized beta = 0.220, *p* = 0.029) PVS volume. Hypertension was positively associated with deep white matter PVS volume (standardized beta = 0.234, *p* = 0.017). Furthermore, we found that ICV was strongly associated with the deep white matter PVS volume (standardized beta = 0.354, *p* < 0.001) while the intracranial artery diameter was negatively associated with the deep white matter PVS volume (standardized beta = −0.213, *p* = 0.032).

**Conclusions:** Intracranial volume has significant influence on deep white matter PVS volume. Future studies on PVS dilation should include ICV as an important covariate.

## Introduction

The perivascular space (PVS) is a major component of the brain glymphatic system (Jessen et al., 2015). Cerebrospinal fluid (CSF) can enter brain parenchyma through PVS, bring metabolic waste out, and maintain brain tissue milieu. During brain aging, the function of PVS may significantly decrease (Benveniste et al., 2019; Francis et al., 2019) due to reduced aquaporin 4 (AQP4) water channels, lower arterial pulsatility force, deposition of amyloid beta (Aβ) proteins, etc. Impaired clearance from PVS may cause aggregation of pathological molecules and further leads to neurodegeneration (Weller et al., 2015).

As more and more evidence suggests the importance of impaired glymphatic function in major brain diseases, it is desirable to assess its function using *in-vivo* imaging methods (Francis et al., 2019). While normal PVS are thin linear structures that can hardly be displayed on clinical brain magnetic resonance images, PVS can remarkably dilate under certain pathological conditions, making it observable (Brown et al., 2018). The potential of using dilated PVS as an imaging marker (Ramirez et al., 2016) for glymphatic dysfunction has been continuously explored. Indeed, PVS dilation has been found associated with Alzheimer's disease (Banerjee et al., 2017), cerebral small vessel disease (Laveskog et al., 2018), stroke (Potter et al., 2015; Zhang et al., 2016), systemic lupus (Miyata et al., 2017), multiple sclerosis (Ge et al., 2005), etc.

To be noted, PVS characteristics exhibit large differences in healthy subjects. Such variations need to be addressed before PVS can be reliably used to reflect pathological changes. A few community-based studies have revealed several determinants of PVS dilation. In the Three-City study, Zhu et al. revealed that PVS dilation was associated with age, hypertension, the volume of white matter hyperintensities (WMH) and the presence of lacunar infarcts (Zhu et al., 2010). Besides, men had severer PVS dilation than women. In the Northern Manhattan Study (NOMAS), age, hypertension, and carotid plaque were found associated with PVS dilation (Gutierrez et al., 2013). In the Kashima scan study (Yakushiji et al., 2014), PVS dilation in the centrum semiovale (CSO) was associated with age, hypertension, lacunar infarcts, and lobar microbleeds; while PVS in the basal ganglia (BG) was associated with sex, hypertension, lacunar infarcts, severer WMH, and subcortical micro-bleeds.

While these community studies generally showed that age, hypertension, and cerebral vascular damages may contribute to PVS dilation, the potential influence of some other factors still remains unclear. In people with large head size, the volume of PVS may be larger due to longer or thicker blood vessels. Because arterial pulsation can drive glymphatic flow and hypertension is associated with PVS dilation, the diameter of blood vessels may also affect this process. Additionally, brain atrophy may create extra space between vessel wall and parenchyma, resulting in larger PVS. The influence of these factors on PVS dilation is yet to be determined. Furthermore, most previous studies used low-resolution clinical images and visual counting methods to evaluate PVS dilation, which may bring bias into the results.

In the present study, we aim to investigate the potential influence of demographic factors, vascular risk factors, intracranial volume (ICV), major brain artery diameters, and brain atrophy on PVS dilation. To achieve good accuracy and stability, we adopted high-resolution multi-modal imaging and machine-learning based segmentation methods, and examined PVS dilation in normal healthy community subjects. We expect similar findings regarding the association between age, hypertension, and PVS dilation. Additionally, we hypothesize that subjects with larger head size, smaller vascular diameters, or more brain atrophy may have more severe PVS dilation.

## Materials and Methods

### Subjects and Clinical Assessments

We searched our prospectively collected imaging database on community subjects (age >50) and included 103 healthy elder subjects. The exclusion criteria include: (1) history of stroke, brain trauma, neurological or psychiatric diseases, nor systematic diseases that could severely affect the brain; (2) metal-implants, claustrophobia, or other inappropriate conditions for MR scans; (3) existence of lacunas, microbleeds, and severe WMH (Fazekas deep or periventricular score > 2), which may heavily influence PVS dilation or bring bias into PVS assessment; (4) cognitive impairment (MMSE < 24), which is likely the result of Alzheimer's or other specific pathologies. Therefore, this cohort was likely to represent a “healthy aging” population, but not a community population with “typical aging.”

All subjects went through a complete assessment of neuropsychiatric conditions, and multi-sequence MRI scans. Hypertension was defined as the presence of any of the following: systolic blood pressure ≥140 mmHg or diastolic pressure ≥90 mmHg measured twice in quiet conditions or having self-reported history of hypertension. Diabetes mellitus was defined as the presence any of the following: fasting serum glucose >7.0 mmol/L or postprandial 2 h plasma glucose >11.1 mmol/L or having previous history of diabetes. Hyperlipidemia was defined as having elevated level of triglyceride, or total cholesterol, or low-density lipoprotein.

### MR Imaging Protocols

All the MR images were acquired using a United Imaging MR790 3.0T scanner. T1 weighted images were acquired with a 3D fast spoiled gradient-echo sequence, the parameters were: TR = 6.9 ms, TE = 2.9 ms, flip angle = 9°, Inversion time = 1,000 ms, field of view = 256 × 240 mm, voxel size = 1 × 1 × 1 mm, 208 sagittal slices. T2 weighted images were acquired with a MATRIX (modulated flip angle technique in refocused imaging with extended echo train) sequence, the parameters were: TR = 3,000 ms, TE = 405.46 ms, echo train length = 180, field of view = 256 × 240 mm, voxel size=0.8 × 0.8 × 0.8 mm, 208 sagittal slices. T2 FLAIR images were acquired with inversion recovery MATRIX sequence, the parameters were: TR = 6,500 ms, TE = 432.48ms, echo train length = 220, bandwidth = 600 Hz/pixel, field of view = 256 × 220 mm, voxel size = 1 × 1 × 1 mm, 170 sagittal slices. A 3D time-of-flight (TOF) magnetic resonance angiography (MRA) sequence was used to assess major brain arteries, the parameters were: TR = 19.1 ms, TE = 4.0 ms, acquisition matrix = 368 × 240, Flip Angle = 16°, voxel size = 0.3 × 0.3 × 0.5 mm, Number of slices = 180. Several other sequences were acquired, and the total scan time was about 1 h.

### PVS Segmentation

We used a deep-learning method to segment PVS. The method was originally developed to segment PVS on isotropic T2 images acquired with 7T scanners, using a novel multi-channel multi-scale fully convolutional network (Lian et al., 2018). To implement this method on our data, 24 additional T2 images acquired using the same protocol were introduced and PVS was hand-depicted by two radiologists who discussed and decided the standard together. These 24 T2 images and the corresponding PVS masks were fed into the neural network to fine-tune the network parameters.

**Figure 1:**
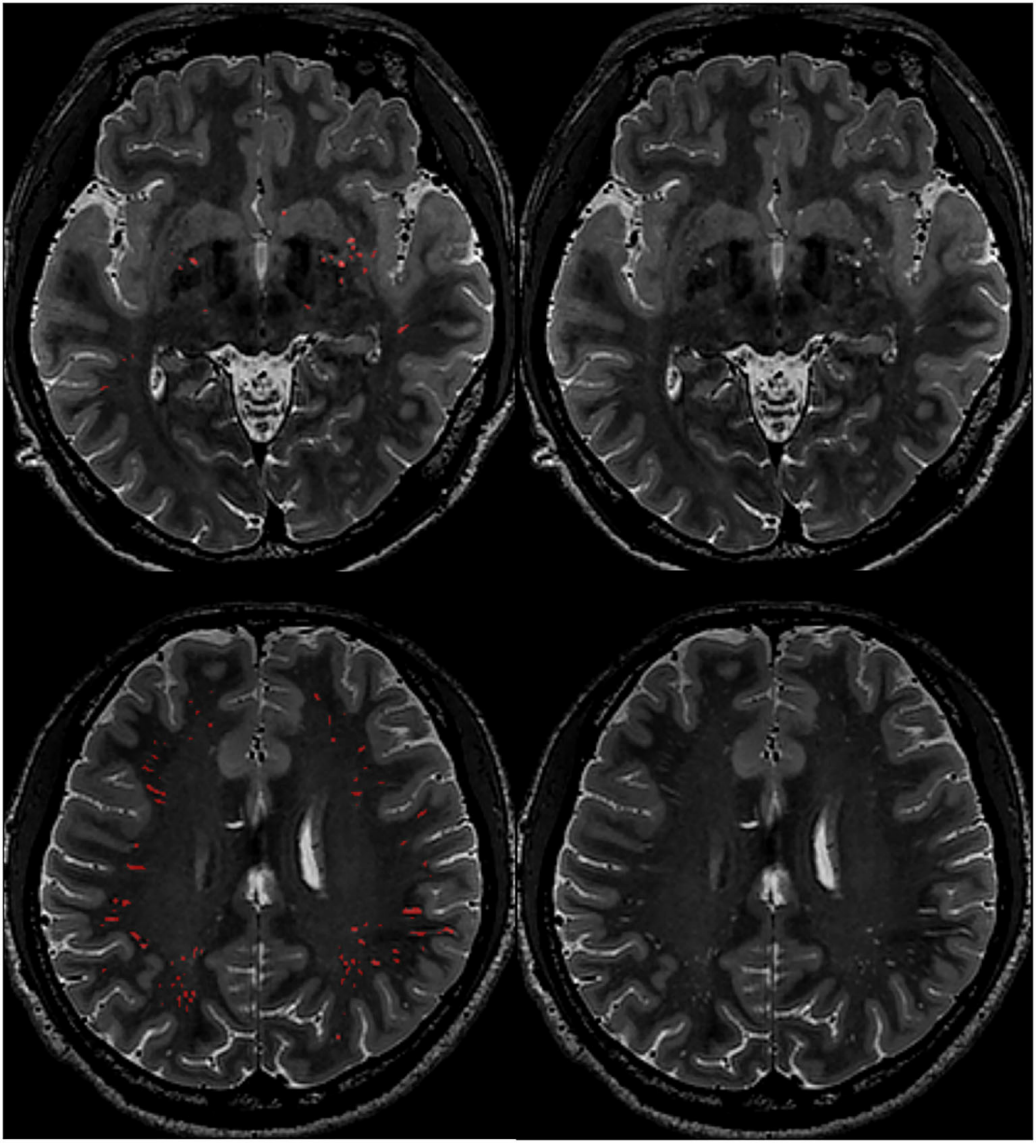
Example images of PVS segmentation results. First row: PVS in the basal ganglia region; Second row: PVS in the deep white matter region. Red color indicates segmented PVS.

Quality assessments were performed to ensure that the neural network had adapted to our data, and PVS could be well-segmented with good accuracy. Specifically, 10 subjects were used to validate the segmentation. The average Dice similarity coefficient, sensitivity, and positive predictive value were 0.71, 0.70, and 0.73, respectively. After validation, all the subjects' PVS were segmented using this method. Finally, one experienced neuroradiologist reviewed all the segmentation results (Figure 1) and manually corrected the masks. For each subject, the PVS mask was checked on axial, coronal, and sagittal planes to ensure that PVS lines were correctly labeled. PVS running at oblique directions and appearing as white dots on orthogonal planes were not added back, because correctly labeling all the small white dots in a slice-by-slice way in over a 100 subjects was almost impossible. Wrongly segmented structures, such as sulcus and WMH, were also corrected. In this way, it would usually take about half an hour to correct one PVS mask.

A ventricle mask was created by dilating the standard brain's lateral ventricles (10 mm out), which was then transformed to each subject's brain space. The BG mask was created by dilating each subject's deep-nucleus segmentation results, which was then used for bgPVS extraction and volume calculation. The deep white matter (DW) PVS volume was calculated in the whole cerebrum after masking out the BG area.

### WMH Segmentation

The WMH segmentation was performed using BIANCA (Brain Intensity AbNormality Classification Algorithm (Griffanti et al., 2016), https://fsl.fmrib.ox.ac.uk/fsl/fslwiki/BIANCA), which is a fully automated, supervised method for WMH detection, based on the k-nearest neighbor (k-NN) algorithm. As BIANCA can be further trained to adapt to different populations, it has higher flexibility and may achieve higher accuracy compared to traditional segmentation methods. We also trained BIANCA with 24 subjects' T2 FLAIR images and depicted WMH masks. To achieve high accuracy, training parameters were also fine-tuned. The final model included intensity information from both T1 images and T2 FLAIR images, as well as the MNI spatial transformation information. We selected 2,000 training points within the WMH area, and 10,000 training points from the normal appearing white matter (NAWM) area. After generating the WMH probability map, we created a mask by dilating the CSF masks inward until reaching the white matter. The mask was applied to the probability map and the results were thresholded by 0.5 to derive the final WMH mask. In general, the segmentation produced robust segmentation results (validated on 10 subjects, DICE coefficient was 0.83). Though, visual assessment and manual correction were still performed to ensure accuracy by an experienced neuro-radiologist.

### Artery Diameter Evaluation

The diameters of the intra carotid artery (ICA) and basilar artery (BA) were measured on the axial slices of 3D-TOF MRA images by a neuroradiologist. Specifically, the ICA was measured at the vertical cavernous segment (Bouthillier et al., 1996; Baradaran et al., 2017; Yeniceri et al., 2017). The BA was measured on the slice at the middle of the pons (Ichikawa et al., 2009; Uceyler et al., 2014). Artery diameters measured on the short axis were used as to avoid oblique effect. Diameters of the bilateral ICA were averaged for further analysis. To assess intra-observer consistency, the neuro-radiologist repeated the procedure on 30 randomly selected cases after 1 month. The intraclass correlation (ICC) index was used to evaluate consistency. Additionally, as stenosis may significantly influence the result, we also checked whether there was stenosis in major arteries. No apparent stenosis was identified in ICA and BA in all subjects.

### Statistical Analysis

As the volume of WMH and PVS were not normally distributed, we performed log-transformation and used logWMH and logPVS in the followed analyses. Firstly, simple linear regression analyses were performed to evaluate the influence of each factor on PVS volume, with bg/dwPVS volume set as the dependent variable and each risk factor set as predictor variable. Standardize beta was used to reflect their predictive ability. Secondly, step-wise Akaike Information Criterion (AIC) regressions were used to perform multiple regression analysis, using the stepAIC function from the “MASS” R package. Forward and backward selection were both performed to select variables. Multi-collinearity analysis was performed using the “VIF” R package. Standardize beta was used to demonstrate each variable's contribution.

## Results

The subjects' characteristics can be seen in Table 1. The mean age was 57.4, ranging from 50.2 to 75.6. There were 56 females (54.4%). The median WMH volume was 1.2 ml (range: 0.7–1.6 ml), and the median PVS volume was 2.6 ml (range: 1.7–3.9 ml). The ICCs demonstrated excellent intra-observer consistency (left ICA ICC = 0.925; right ICA ICC = 0.967, BA ICC = 0.948).

**Table 1:** Characteristics of the study participants.

**Characteristics**	***N*** **= 103**
Age, y, mean ± SD	57.4 ± 6.1
Women, *n* (%)	56 (54.4)
Education, y, mean ± SD	8.3 ± 3.3
Hypertension, *n* (%)	37 (35.9)
Hyperlipidemia, *n* (%)	19 (18.5)
Diabetes, *n* (%)	12 (11.7)
Smoker, *n* (%)	30 (29.1)
MMSE, mean ± SD	28.0 ± 2.5
PVS volume, ml, median (interquartile range)	2.6 (1.7–3.9)
WMH volume, ml, median (interquartile range)	1.2 (0.7–1.6)
ICA diameter, mm, mean ± SD	3.8 ± 0.4
BA diameter, mm, mean ± SD	2.7 ± 0.5
Intracranial volume, mL, mean ± SD	1,523.7 ± 1,480.0
Brain to ICV ratio, %, mean ± SD	73 ± 2.9
Ventricle to ICV ratio, %, mean ± SD	1.5 ± 0.6

*PVS, perivascular space; WMH, white matter hyperintensity; ICA, intra-cerebral artery; BA, basilar artery; ICV, intracranial volume*.

**Figure 2:**
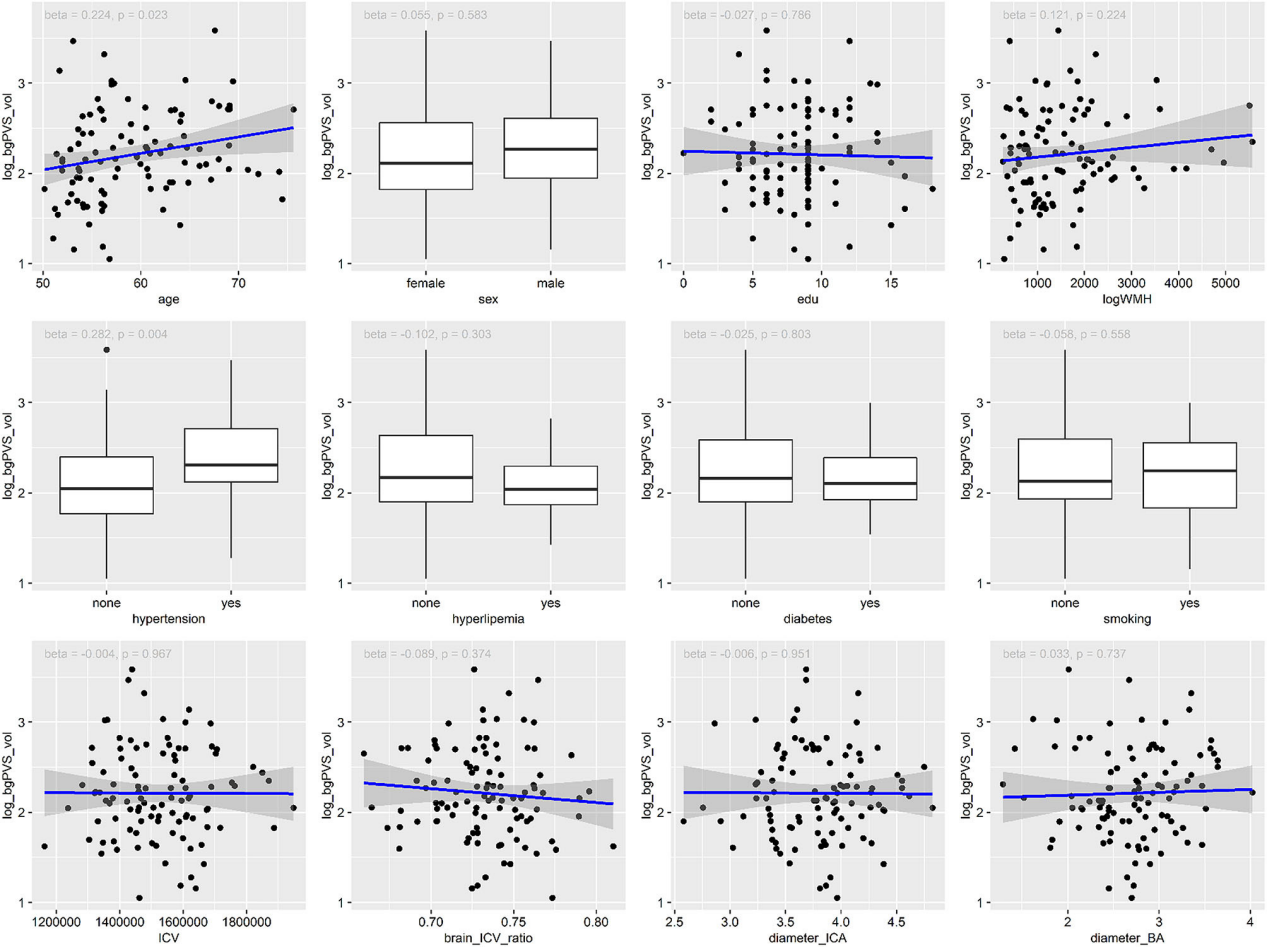
Association between risk factors and the volume of bgPVS.

**Figure 3:**
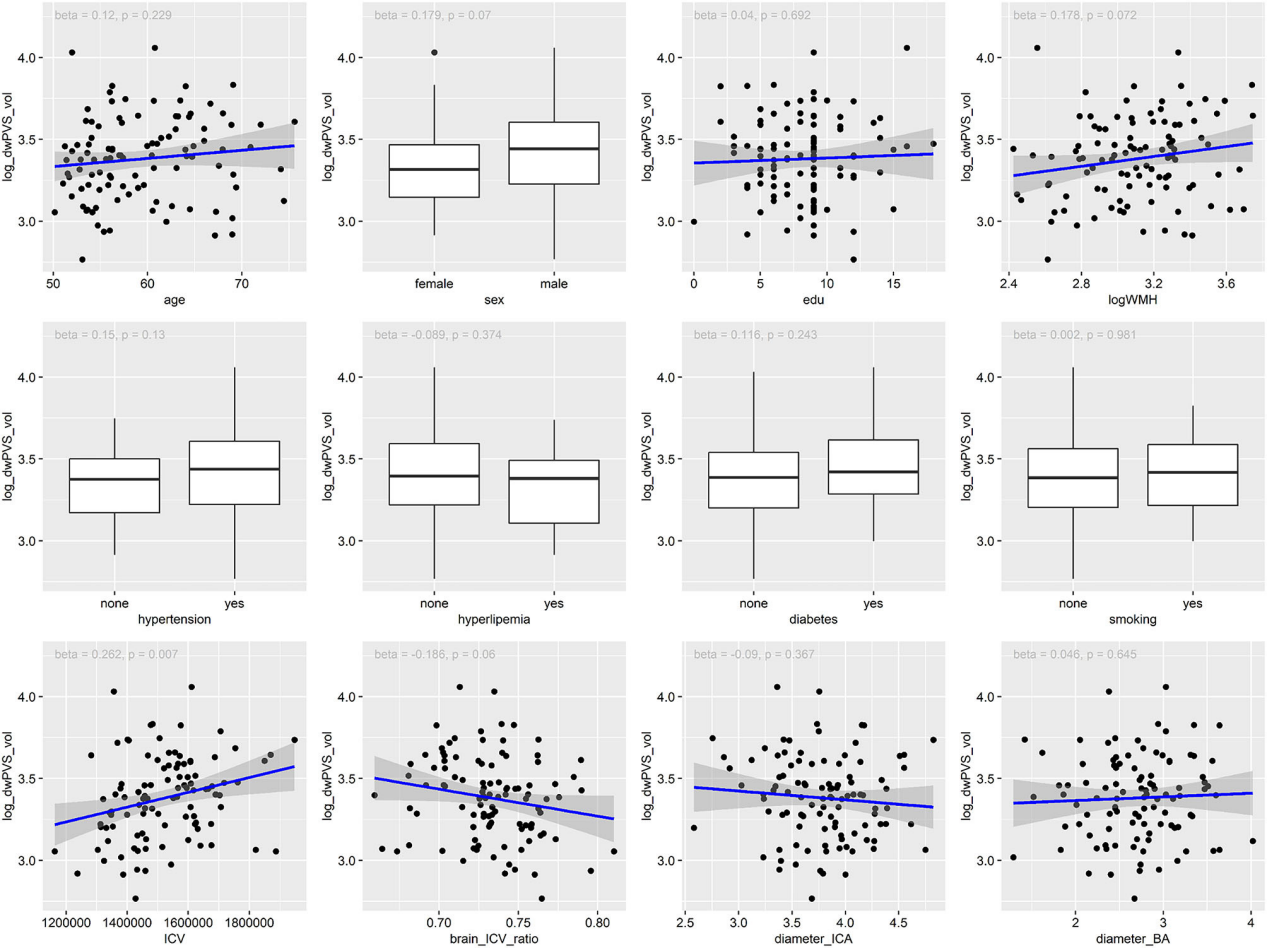
Association between risk factors and the volume of dwPVS.

As shown in Table 2, Figures 2, 3, Univariate regression analysis showed that age and hypertension were significantly associated with bgPVS volume, while ICV was significantly associated with dwPVS volume. The associations between dwPVS and sex, logWMH, brain_ICV_ratio were marginally significant.

**Table 2:** Results of simple linear regression analyses.

	**Deep white matter PVS**		**Basal ganglia PVS**	
	***β-*****Coefficient^**#**^**	***p*****-Value**	***β-*****Coefficient^**#**^**	***p***-**Value**
Age	0.120	0.229	0.224	0.023*
Sex	0.179	0.070	0.055	0.583
Education	0.040	0.692	−0.027	0.786
Hypertension	0.150	0.130	0.282	0.004*
Diabetes	0.116	0.243	−0.025	0.803
Hyperlipidemia	−0.089	0.374	−0.102	0.303
Smoking	0.002	0.981	−0.058	0.558
ICA diameter	−0.090	0.367	−0.006	0.951
BA diameter	0.046	0.645	0.033	0.737
WMH volume (log-transformed)	0.178	0.072	0.122	0.224
Intracranial Volume	0.262	0.007*	−0.004	0.967
Brain to ICV ratio	−0.186	0.060	−0.089	0.374

# Standardized β; ICA, intra-cerebral artery; BA, basilar artery; WMH, white matter hyperintensity; ICV, intracranial volume. Bold values and * indicate a p < 0.05.

Multi-collinearity analysis showed low interaction among the included variables (VIF: 1.05–2.66). Multiple regression analysis showed that age and ICV were positively associated with dwPVS volume (Table 3), while hyperlipidemia and ICA diameter were negatively associated with dwPVS volume. On the other hand, age and hypertension were positively associated with bgPVS volume, while hyperlipidemia was negatively associated with bgPVS volume (marginally significant).

**Table 3:** Results of multiple regression analyses.

	***β-*****Coefficient^**#**^**	***p*****-Value**
**Deep white matter PVS**, ***adjusted R**^**2**^**=0.123, p=0.002***		
Age	0.220	0.029
ICV	0.354	<0.001
hyperlipidemia	−0.215	0.033
ICA diameter	−0.213	0.032
**Basal ganglia PVS**, ***adjusted R**^**2**^**=0.108, P=0.002***		
Age	0.227	0.027
Hypertension	0.234	0.017
Hyperlipidemia	−0.175	0.078

# *Standardized β; PVS, perivascular space; ICV, intracranial volume; ICA, intra-cerebral artery*.

To help interpreting ICA's contribution, we performed additional analyses and found significant correlation between ICA diameter and ICV (Pearson's *r* = 0.258, *p* = 0.008), and significant correlation between ICA diameter and WMH volume (Pearson's *r* = 0.168, *p* = 0.003) after controlling age, sex, and ICV. Furthermore, subjects with hypertension had larger ICA diameters (mean = 3.882) than those without hypertension (mean = 3.733 mm, *p* = 0.08).

## Discussion

In the present study, we investigated the influence of a variety of risk factors on PVS dilation. We employed high-resolution multi-modal imaging acquisition and machine-learning based analytical methods, and confirmed previous findings that age and hypertension were positively associated with PVS dilation. Furthermore, we found that ICV was positively associated dwPVS volume while ICA diameter was negatively associated with dwPVS volume. These findings may help us better understand the mechanism of PVS dilation, and guide future PVS studies by specifying confounding factors.

Age was found associated with both bgPVS and dwPVS, which has been reported in previous studies, although the association may vary due to sample characteristics and evaluation methods. For example, people older than 65 had more dilated PVS in BG but not DW areas (Zhang et al., 2014). Age was associated with BG but not DW PVS scores in the NOMAS study (Gutierrez et al., 2013), and in the Rotterdam Study (Dubost et al., 2019). On the other hand, some studies also suggest that BG & DW PVS were both associated with age (Zhu et al., 2010). The relationship between hypertension and PVS was also similar to previous studies. More studies reported stronger association between hypertension and bgPVS than dwPVS (Zhu et al., 2010; Gutierrez et al., 2013; Martinez-Ramirez et al., 2013; Yakushiji et al., 2014; Dubost et al., 2019), except for Zhang's study (Zhang et al., 2014). The difference between BG and DW PVS could be due to their distinct anatomical structures, as well as factors such as artery diameters and blood pressure (Brown et al., 2018).

As hypothesized, we found positive correlation between ICV and dwPVS. Intracranial volume was the strongest predictor in multiple regression analysis (beta = 0.354, *p* < 0.001). Apparently, in people with large heads, longer or thicker blood vessels (van der Zwan et al., 1993) are needed to maintain brain blood supply, which allows more PVS dilation during brain degeneration. However, such association has often been neglected in previous studies. Although most previous studies used low-resolution MR images and visual rating scores instead of quantitative methods, ICV could still influence the results, because a recent study showed that results derived from automated segmentation or visual assessment were highly correlated (Dubost et al., 2019).

Previously, several studies reported that males tend to have more PVS dilation than females. For example, the Sunnybrook Dementia Study found that men had severe dwPVS dilation than women in both Alzheimer's disease patients and normal controls (Ramirez et al., 2015). Similarly, Zhang et al. found that men had more PVS dilation in white matter and hippocampus (Zhang et al., 2014). The Three-City study also found greater PVS dilation in men relative to women, although it was bgPVS rather than dwPVS that showed differences (Zhu et al., 2010). As the reason for sex difference on PVS dilation was still unclear, Ramirez et al. suggested that anatomical differences in PVS structures, the potential effect of astrocytic response to inflammation and hormonal interactions with the glymphatic systems might be the contributing factors (Ramirez et al., 2016). In our study, although we found that men had larger dwPVS volumes than women in univariate analysis, the effect disappeared in multivariate regression analysis. Considering that men usually have larger head than women (in this study, men's mean ICV: 1,620,657 mm^3^; women's mean ICV: 1,442,351 mm^3^), we infer that the difference of PVS volumes between men and women are likely due to different head sizes. Therefore, when ICV was included in multiple regression analysis, sex no longer made significant contribution to PVS dilation.

We found negative association between ICA diameter and dwPVS. Additionally, ICA diameter was associated with a variety of physiological and pathological factors, including ICV, WMH volume, and hypertension. Therefore, its association with PVS volume is also a complicated issue. While larger head size, higher age, and hypertension might all lead to larger ICA diameter, controlling their effects during multiple regression analysis yielded negative association between ICA diameter and dwPVS. We speculate that this could be due to physiological mechanisms. With the same head size, smaller artery diameters may create larger pulsatility force that pushes through brain parenchyma, thus generate more PVS. This inference need to be tested in future studies.

The association between WMH and dwPVS, and the association between brain_ICV_ratio and dwPVS were marginally significant in univariate analysis. Indeed, it has been demonstrated that various pathologies in cerebral small vessel disease may contribute to PVS dilation (Zhu et al., 2010; Gutierrez et al., 2013; Del Brutto and Mera, 2017), and ex-vacuo dilatation secondary to the shrinkage of brain parenchyma may create more PVS (Zhang et al., 2016). However, these correlations did not survive in multivariate analysis. This might be due to population characteristics. Here we selected healthy elderly subjects, who had very mild WMH (median WMH volume = 1.2 mL, Fazekas deep white matter score or periventricular score <2), and were relatively younger than other community studies (mean age = 59.5, compared to 71.6 in NOMAS or 72.5 in Three-city study). We also excluded those with cognitive impairment to avoid possible influence from other pathological factors. Therefore, the effect of vascular degeneration, brain atrophy, and other pathological sources was mild, thus the influence of some basic demographic factors could be highlighted.

In a recent study using brain MRI scans from the Rotterdam Study, Florian et al. developed a new algorithm for automated quantification of PVS (Dubost et al., 2019). They found that age, sex, blood pressure, glucose, lacunar infarcts, cortical infarcts, and white matter hyperintensity (WMH) were associated with BG-PVS volume; on the other hand, sex, body mass index, lacunar infarcts, ApoE4, and ICV were associated with CSO PVS volume. While their study showed similar results with the present one, they mainly focused on the development of segmentation techniques and had not performed multiple regression analysis, making it difficult to understand the true contribution of each predictors and to interpret those findings.

The current study is subject to limitations. First, there are many ways of measuring arterial diameters. Some researchers measured on 3D volume-rendering images while others preferred axial images, and the measurement locations also varied from study to study. As each of the method has its own advantages and disadvantages, there is still no consensus yet. Second, we had not examined the influence of genetic mutation on PVS dilation, which had been implicated in previous studies (Duperron et al., 2018; Dubost et al., 2019). Third, the sample size was moderate compared to some previous clinical studies. However, as healthy subjects usually have less inter-subject variations and we adopted quantitative methods, the results were relatively robust and consistent with previous studies.

In conclusion, we acquired high-resolution brain images and analyzed the association between a variety of risk factors and PVS dilation in healthy elderly subjects. We confirmed the association between age, hypertension, and PVS dilation. Additionally, we found significant association between ICV, ICA diameter, and PVS volumes. We argue that that these factors need to be taken into consideration in future clinical PVS studies.

## Data Availability Statement

The data supporting the findings of this study are available from the corresponding author upon reasonable request. They are not publicly available because of ethical restrictions.

## Ethics Statement

The studies involving human participants were reviewed and approved by the medical ethics committee of the Second Affiliated Hospital, Zhejiang University School of Medicine. The patients/participants provided their written informed consent to participate in this study.

## Author Contributions

PH and MZ were responsible for the study concept and design. YJ, SW, HH, KL, QZ, XL, WY, XW, XX, and XY contributed to the acquisition of imaging data. PH, ZZ, RZ, YJ, and CL performed data analysis and interpreted the findings. PH and ZZ drafted the manuscript. MZ and YY provided critical revision of the manuscript for important intellectual content. All authors contributed to the article and approved the submitted version.

## Conflict of Interest

The authors declare that the research was conducted in the absence of any commercial or financial relationships that could be construed as a potential conflict of interest.
